# Correction: Asymmetric catalytic [1,3]- or [3,3]-sigmatropic rearrangement of 3-allyloxy-4*H*-chromenones and their analogues

**DOI:** 10.1039/d4sc90148g

**Published:** 2024-08-02

**Authors:** Yi Li, Lichao Ning, Qi Tang, Kexin Lan, Bingqian Yang, Qianchi Lin, Xiaoming Feng, Xiaohua Liu

**Affiliations:** a Key Laboratory of Green Chemistry & Technology, Ministry of Education, College of Chemistry, Sichuan University Chengdu 610064 China liuxh@scu.edu.cn xmfeng@scu.edu.cn

## Abstract

Correction for ‘Asymmetric catalytic [1,3]- or [3,3]-sigmatropic rearrangement of 3-allyloxy-4*H*-chromenones and their analogues’ by Yi Li *et al.*, *Chem. Sci.*, 2024, **15**, 11005–11012, https://doi.org/10.1039/D4SC02201G.

The authors regret that an important reference that should be cited was missed in the original article; this reference is shown below as ref. 4. The corresponding revised text is as follows.

There are rare examples related to asymmetric catalytic C2-functionalization of 3-hydroxychromenones:^1–4^ one is a chiral Pybox–Sc(iii)-complex-catalyzed formal [3,3]-rearrangement to construct 3,4-chromanediones by Porco and co-workers,^1^ and the other is chiral NHC-initiated formation of an α,β-unsaturated acyl azolium intermediate to perform Coates–Claisen rearrangement by Bode's group^2^ and Rafiński's group.^3^

Furthermore, other important compounds derived from kojic acid (**5q**), allomaltol (**5r**) and lawsone (**5s**) could be efficiently constructed in good yields (64–87%) and enantioselectivities (74–92% ee). These products were previously obtained *via* iridium-catalyzed allylic alkylation by Mukherjee *et al*.^4^

The Royal Society of Chemistry apologises for these errors and any consequent inconvenience to authors and readers.

## References

[cit1] Maríe J., Xiong Y., Min K., Yeager A. R., Taniguchi T., Berova N., Schaus S. E., Jr J. A. Porco (2010). J. Org. Chem..

[cit2] Kaeobamrung J., Mahatthananchai J., Zheng P., Bode J. W. (2010). J. Am. Chem. Soc..

[cit3] Dzieszkowski K., Słotwiński M., Rafińska K., Muzioł T. M., Rafiński Z. (2021). Chem. Commun..

[cit4] Mitra S., Sarkar R., Chakrabarty A., Mukherjee S. (2022). Chem. Sci..

